# Space hardware for concrete sample production on ISS “MASON concrete mixer”

**DOI:** 10.1038/s41526-023-00304-0

**Published:** 2023-07-21

**Authors:** J. T. I. Müller, B. Rattenbacher, K. Tell, C. Rösch, T. Welsch, M. Maurer, M. Sperl, M. Schnellenbach-Held

**Affiliations:** 1 University of Duisburg-Essen (UDE)—Institute for Structural Concrete, Essen, Germany; 2 Lucerne University of Applied Sciences and Arts (HSLU)—BIOTESC, Lucerne, Switzerland; 3 German Aerospace Center (DLR)—Institute of Materials Physics in Space, Cologne, Germany; 4 European Space Agency (ESA)—European Astronaut Centre, Cologne, Germany; 5 University of Cologne (UoC)—Institute for Theoretical Physics, Cologne, Germany

**Keywords:** Techniques and instrumentation, Engineering

## Abstract

Advances in space flight technology will enable the construction of Moon or even Mars bases in the not-too-distant future. Thus, materials will be needed that are suitable for building in microgravity environments. One idea is to use concrete, the most used construction material on Earth, for these challenging tasks. The hardening and the properties of concrete under the boundary conditions prevailing on Earth are well understood, but there is only limited research on concrete produced in microgravity. Hence, a research project called MASON was established, which aims to mix and harden concrete on the ISS and to investigate the properties of the specimens made in microgravity extensively. Since a defined geometry of the specimens would be favorable for these investigations, a special hardware was developed, called the MASON Concrete Mixer (MCM), which allows the production of concrete specimens fulfilling the requirements on the geometry as well as the safety requirements. Subsequently, the development, design, tests, and qualification of the MCM as well as its usage are presented.

## Introduction

In the past decades, the material properties of cement paste and solid concrete have been extensively researched within the boundary conditions prevailing on Earth and are thus well understood^1^. Continuous advances in space flight technology allow increasingly easier access to space and planetary surfaces^2,3^. To explore these surfaces, building materials are required in large quantities in order to build space infrastructure or future Moon or Mars bases^4–6^. Accordingly, it is more important than ever to investigate and understand the behavior of building materials under the conditions prevailing in space^7^. This especially holds for cement-based concrete, the most used construction material on Earth. Although the use of cementitious binders on celestial bodies is rather unlikely due to efforts that would be necessary for the transport, a deep understanding of the effects of microgravity on hardening and mechanical properties of concrete will provide general knowledge for the use of mineral construction materials in space. However, the investigations on the properties of cement-based composite materials hardened under microgravity are fairly limited thus far. Some work has been published on the crystallization of cement under weightlessness, in short duration during parabolic flights^8–12^ as well as in long-term^13,14^, and only one single experiment on mixtures of cement, sand and liquids: Already in 1994, first investigations were performed onboard Space Shuttle Endeavor (STS-68). However, the “ConCIM”-experiment was only successful to a limited extent, since the single sample that was produced exhibited a poor quality due to a hardware failure^15–17^.

In the MASON (“**MA**terial science on **S**olidification of c**ON**crete”) project group, the influence of microgravity on the solidification and properties of concrete mixed and hardened in space was studied. For this investigation, an experiment on the International Space Station (ISS) was carried out. Simultaneously, analogous experiments were conducted on Earth to obtain reference samples hardened under Earth gravity. Additionally, concrete specimens using clinostats and random positioning machines (RPM) were produced to achieve insights in the hardening of concrete under simulated microgravity.

**Fig. 1:**
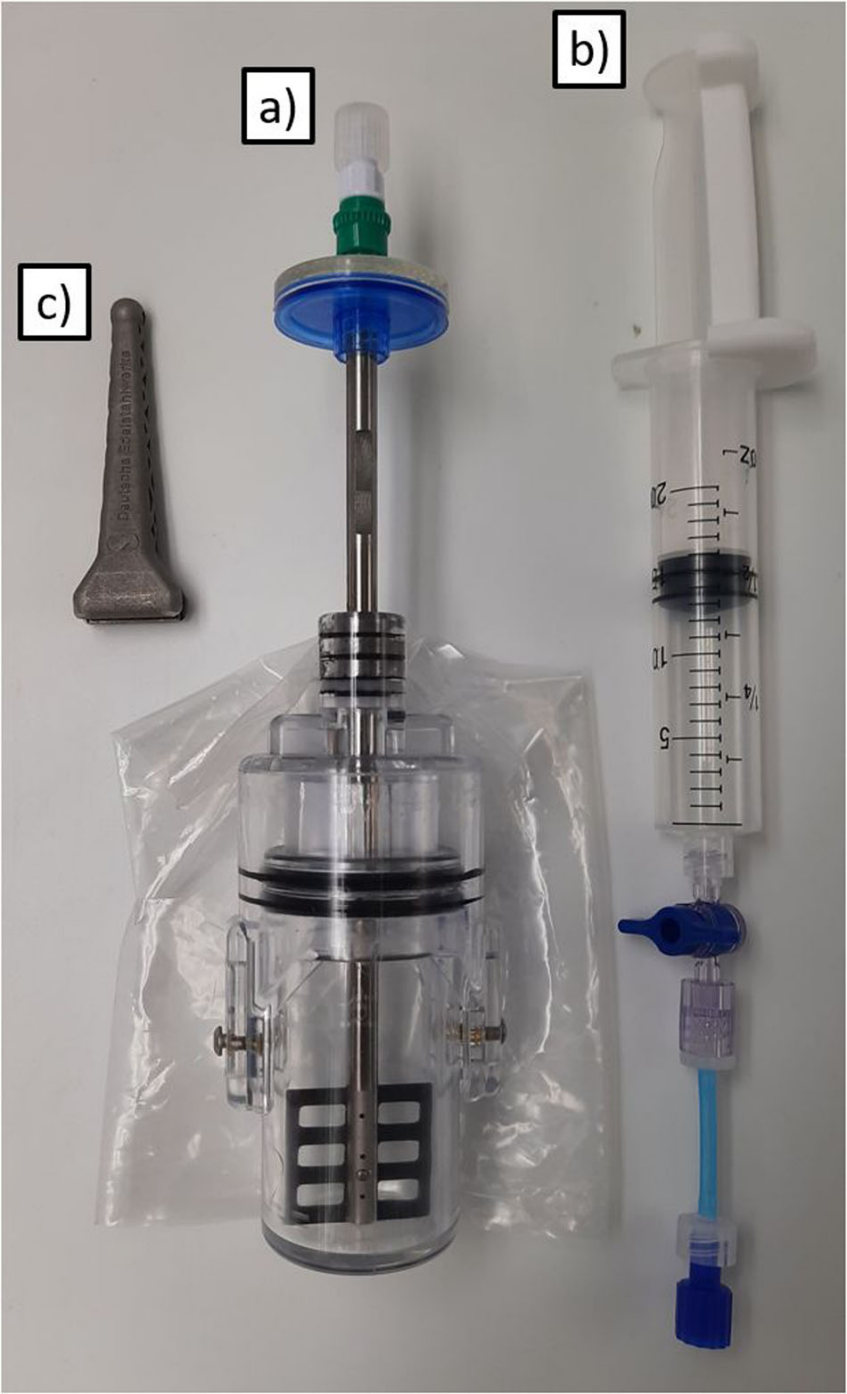
Items of a MASON Kit. **a** MASON Concrete Mixer, **b** syringe and **c** crank.

On behalf and under the guidance of the teams from UDE, UoC, BIOTESC and DLR, the German ESA Astronaut Matthias Maurer produced a total amount of 64 concrete samples on the ISS. To achieve this, a special hardware, called “MASON Concrete Mixer” (MCM), was designed which meets the requirements for a preferably easy experiment execution and for safety on board the ISS (see Fig. 1). In addition, it also had to fulfill the requirement of producing concrete samples with a cylindrical shape, which are suitable for the determination of the microstructure, the air pore distribution, and the mechanical properties.

After initial feasibility studies, the design of the MCM was developed within 9 months in an elaborate process involving the Institute of Structural Concrete (ISC, University of Duisburg-Essen, Germany), BIOTESC (Lucerne University of Applied Sciences and Arts, Switzerland), Institute for Theoretical Physics (University of Cologne, Germany) and the German Aerospace Center - Institute of Materials Physics in Space (DLR, Germany). After the final design of the MCM was found, the experimental and numerical investigations were carried out which were required for the certification of the flight hardware by the ESA safety panel. Once the certification by ESA was obtained, the 64 MCM items were prepared and delivered just in time in autumn 2021 for the upload with SpaceX 24, one year after beginning of the project. In February 2022, Matthias Maurer conducted the experiments on the ISS, where the specimens were stored for planned download on SpaceX 25. Subsequently, the requirements on the MASON hardware, the process of development and qualification, the mixing process on ground and on orbit as well as the specimens obtained using the MCM are presented.

The aim was to obtain a homogeneous concrete specimen with a defined geometry and volume as a baseline for the studies. To produce concrete, the dry cement powder and aggregates must be mixed with water within the weightless environment of the ISS by an astronaut. This environment is very special, as powder, when released, will remain in the air and will be distributed throughout the station by the ventilation system. Cement powder is considered irritating to the skin and the respiratory tract and dangerous when it gets in contact with the eyes. Because of this reason, the cement powder was classified by the NASA mission toxicologist as a "Toxicity Hazard Level 2" (THL 2). The major challenge now was to generate a hardware that fulfills the safety standard of containing this THL 2 substance in all phases of the hardware cycle. It was necessary to integrate 3 independent levels of containment (LOC) into the hardware or produce a design for minimum risk (DFMR). In conclusion, the aspects presented in Table 1 had to be considered in the design of the space hardware. Furthermore, the requirements from NASA^18,19^ had to be considered and served as a base for the hardware development.

**Table 1:** Safety and scientific hardware requirements.

Safety requirements	Scientific requirements
LOC for cement powder and fresh concrete THL 2: Provide 3 LOC for MCM.	Shape and quality Obtain a homogeneous concrete specimen with a defined geometry and volume: Adjustable axial size of container after mixing to compress fresh concrete and ensure same volume for space and reference ground samples.
Stress resistance Sturdy, pressure and vacuum tight, withstanding stresses resulting from rocket launch, pressure drop during flight and operation on ISS by astronaut.	Exact composition of ingredients Addition of water (and additive, if necessary) at a selected time as centrally as possible to dry and loose stored ingredients to ensure good mixability, no loss of water and exact composition of ingredients.
Chemical stability Alkaline fresh cement: Resistance to basic substances.	Control during operation Transparent container: Control homogenous mixture of components inside container during mixing process.
LOC for additives Additive with THL 1: Provide 2 LOC for syringe.	Mixing mechanics Extraction of mixer with thin and optimal performing mixer blade out of concrete sample after mixing: second chamber to store mixer blade after mixing.
Inspection and control Transparent container: Inspection of built-in parts and connections after assembly.	Exhaustion To ensure mixing quality by astronaut: Design of a mixing tool to facilitate mixing.
Reference tests and batch qualification Batch production of hardware to ensure same quality of all MCMs: tests from a representative number of containers are valid for all.	Quantity Minimum of 3 samples of one mixture and three times the amount for reference ground samples: Affordable batch production of MCMs. Keep upload and download costs as low as possible, requiring a lightweight and small construction.
Mixing tool Design of sturdy mixing tool: No exertion of excessive forces on the hardware.	Extraction Extraction of the specimen without damage: container with smooth surface and light inclination.

## Results

### Development of the MCM

The MCM consists of 18 standard parts and only five custom parts, optimized for the intended use. The design drawings of the custom parts formed the basis for the prototype construction using 3D printing. The design was adapted, tested, improved, and printed again multiple times. The final container shape was later produced by injection molding.

**Fig. 2:**
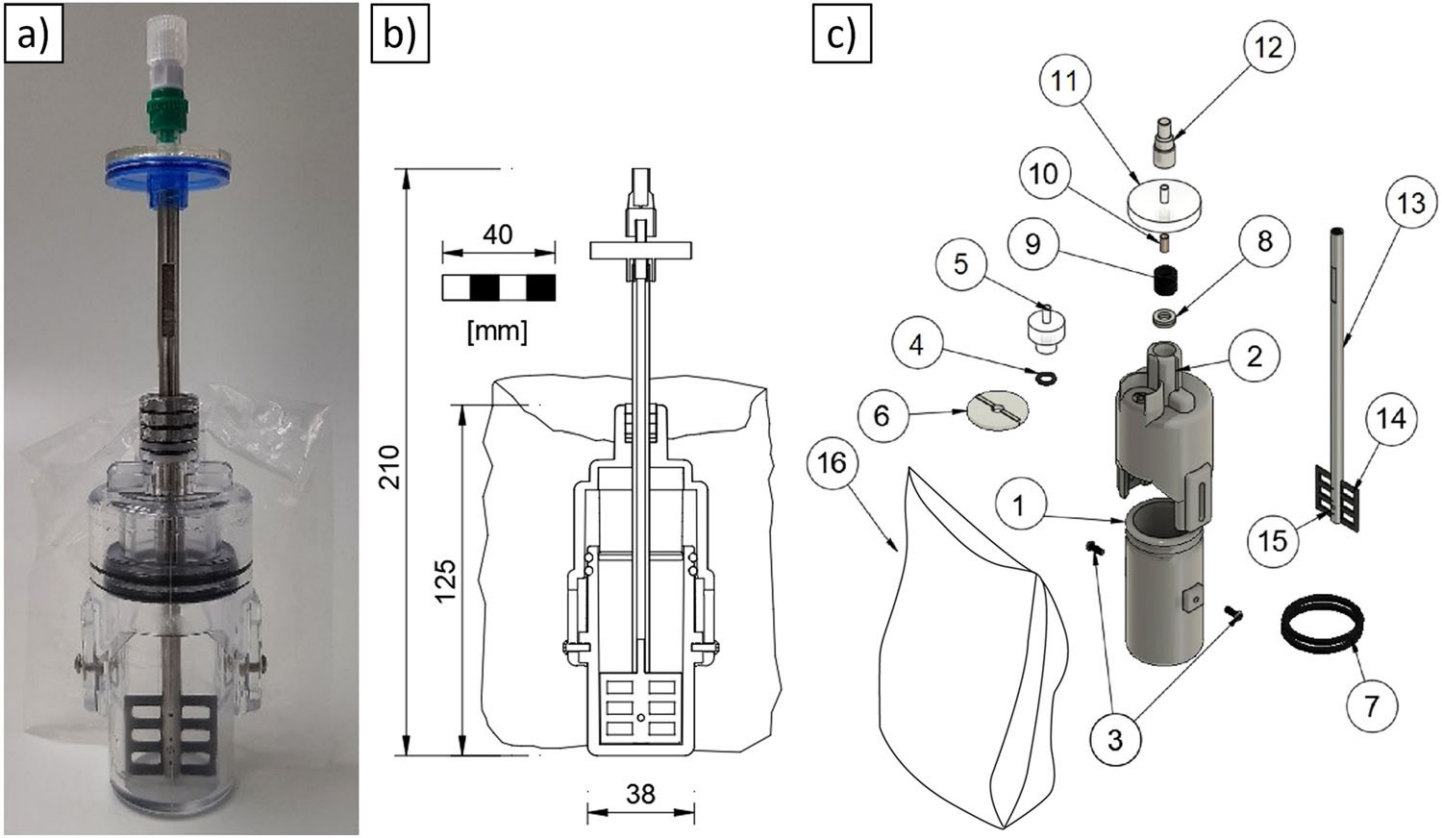
Design of the Mason Concrete Mixer (MCM). **a** overview, **b** cross section view and **c** exploded drawing.

The idea was, as already hinted at in the requirements above, to build a container with 3 main parts: The lower part, a cylinder that holds the cement and later the concrete sample, called the mixing chamber (1, see Fig. 2 for reference to part numbers). The upper part, later called the retention chamber (2), which allows compression of the sample after mixing and the retention of the mixer. And the mixer itself (13) to stir the concrete; this mixer has to protrude the retention chamber, and for mixing, it needs to be turned and later on removed from the fresh concrete.

The mixing and the retention chamber are inserted into each other. These two parts are connected by two opposing screws (3) which are inserted in a slotted hole on the retention chamber and threads on the mixing chamber. In between the walls of the two chambers two O-rings (7) are placed to ensure tightness at the contact surface. To allow compression of the concrete, the retention chamber is equipped with a cylindrical plug, which protrudes into the mixing chamber. This plug will compress the concrete mixture by reducing the volume of the mixing chamber when the mixing and the retention chamber are pressed together. The maximum way of compression is defined by the slotted hole on the retention chamber. By compression of the two chambers pressure will build up. This pressure is released through a hydrophobic filter (5), which on one hand retains the cement powder and on the other hand also the water and liquid basic solution that is generated by adding water to the cement. This filter is additionally enclosed in a PE bag (16) with a volume of approx. 200 ml which acts as a safeguard in case something would escape the hydrophobic filter. The retention chamber gets protruded by the shaft of the mixer. The protrusion is equipped with three shaft seals (9) and two scrapers (8) that allow rotation around the rod axis for mixing the concrete and extraction from the concrete into the retention chamber while at the same time ensuring the leak proofness. This shaft also serves as an inlet pipe for the water or additives. The shaft is a hollow tube made of stainless steel, where a carbon mixer blade^20^ (14) is inserted into a slot on one end. The mixer blade is glued and additionally connected by a bolt (15). After mixing, the mixer blade can be pulled up and stored in the retention chamber to not interfere with the sample. Initial mixing tests have shown that, particularly when sand is added, a high force is required to rotate the tube together with the mixer blade. To counteract this, a special tool (MASON Crank, Fig. 1c) was printed from stainless steel 316L^21^. It gets connected to groves on the mixer shaft and facilitates mixing by providing an adequate lever. At the upper end of the tube a hydrophilic filter (0.45 µm, 11) is connected followed by a check valve (12). Filter and thread both contain Luer Lock connectors. The Luer system is used as a uniform standard in medical technology for the connectivity of infusion sets and syringes and was already approved as space hardware for an experiment on behalf of the student laboratories of the German Aerospace Center (“DLR_School_Lab”) within the mission “ISS Horizons”^22^. The syringe can later be connected to the check valve. The connection is closed with a cap for transport. The entire construction weights approx. 137.5 g and is shown in the final drawing (see Fig. 2).

To mix concrete, water or additive must be provided. This second element is realized with standard medical grades syringes (see Fig. 1b), which are filled with the required amount of liquid. To prevent the plunger from slipping of the syringe, it is held in position by a printed polycarbonate clamp. A one-way valve is attached to the syringe via a Luer thread for opening and closing. This is in turn connected to a short tube, which can later be connected to the container via a Luer adapter. All parts that are to be connected immovably and permanently are screwed together via a Luer-thread and additionally glued with Loctite 4902^23^.

### Safety considerations and LOC philosophy

**Fig. 3:**
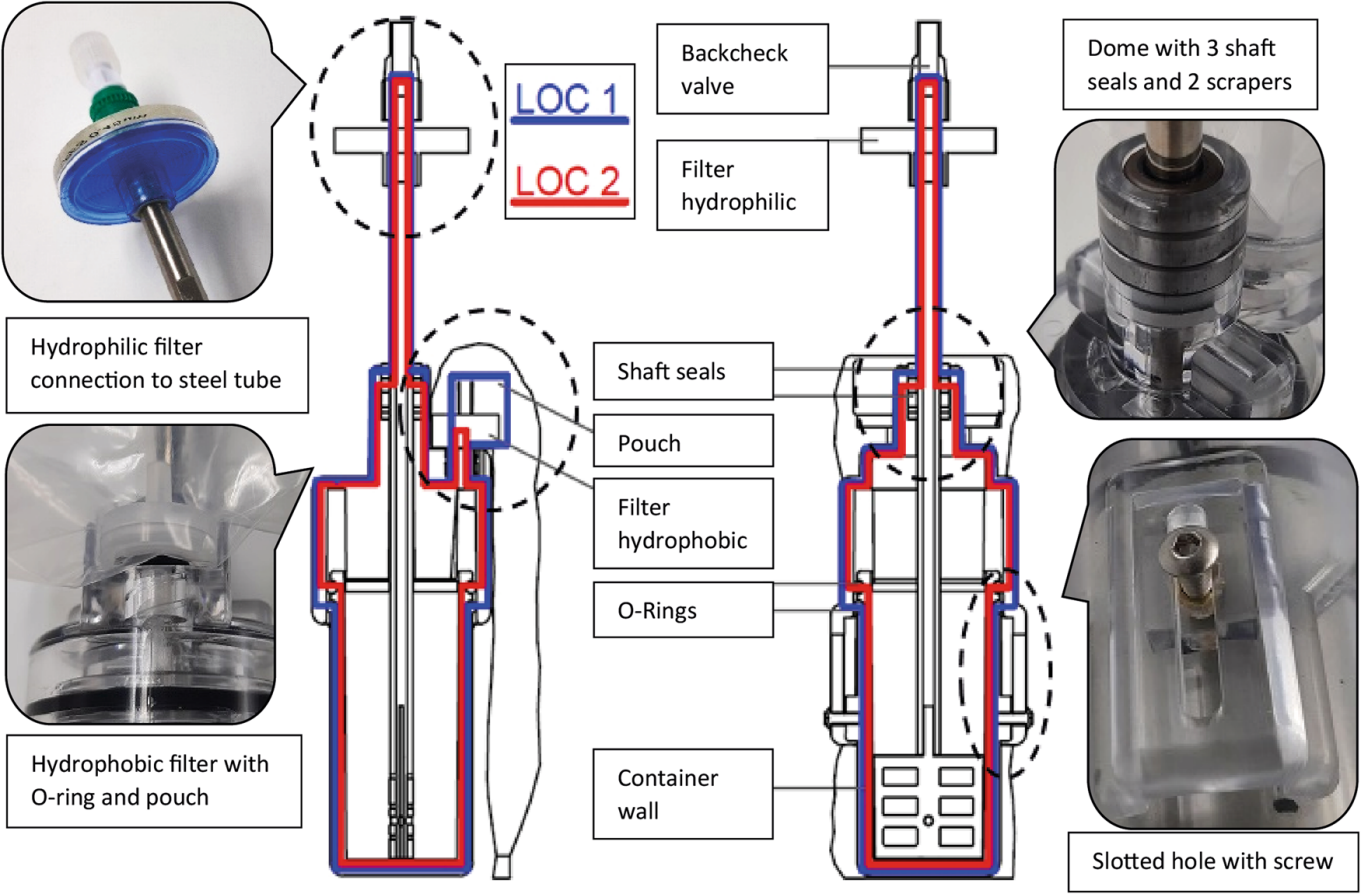
LOC philosophy and details of the MCM. Side and front view of the MCM with display of the LOC (LOC 1 = blue, LOC 2 = red). Close-ups of the construction details of the four circled areas are shown.

As mentioned above, the MCM shall be used on the International Space Station. This special environment concerning microgravity and the limited volume of the ISS poses particular challenges to the hardware design. As it was intended, in the best circumstances, to achieve three independent levels of containment, a construction was strived that combines a design for minimum risk (DFMR) approach with the level of containment (LOC) approach (Fig. 3). The mixing chamber, the retention chamber, and the mixing tube itself followed the DFMR approach. For the retention chamber polycarbonate^24^ with a wall thickness of 4 mm was chosen. Polycarbonate in this setup is able to withstand high mechanical forces and is almost impossible to break. The retention chamber performed as well in this configuration, but when liquid cement got in contact with the complex architecture of the upper part of the retention chamber, environmental stress cracks began forming. Following this issue, the material was reconsidered. CoC and Tritan MX731^25^, a special kind of PET, were tested. Retention chambers produced with CoC were not satisfactory, Tritan MX731 performed well and was chosen. The shaft of the mixer is also part of this DFMR and in this function has to withstand the shear forces during mixing, forces of leverage, when the shaft is moved out and angular forces that could be applied by twisting the MASON crank. At first a wrapped carbon tube was tried, but it sheared already at a force of 1 Nm. It was replaced by a tube made of chemical resistant stainless steel 316L^26^ with 1.5 mm wall thickness. Also part of the DFMR are the connection of the hydrophilic filter to the mixing shaft and the hydrophobic filter to the retention chamber. The hydrophilic filter is screwed into the mixing tube. For this a thread was cut into the Luer Slip of the hydrophilic filter. The filter was then screwed in place and fixed with instant glue. The gap between the Luer Male and the shaft were potted with a two-component epoxy filler^27^ (Fig. 3, upper left). This configuration turned out to be very stable. The hydrophobic filter was screwed into a Luer connector that was cast into the retention chamber (Fig. 3, lower left). On top it was, as above, glued with instant glue and potted with the epoxy filler.

The LOC philosophy was applied, where the parts join, or where the container interacts with the environment. The connection of the mixing chamber and retention chamber was sealed by two O-rings, where each O-ring provides one LOC. The hydrophobic filter (Fig. 3, lower left) allows release of air, which is important when adding the liquid or compressing the mixer. Here the LOC are established by the filter membrane and a plastic bag that covered the filter. The hydrophilic filter on top of the shaft of the mixer is somewhat special in this respect, as the housing of the filter is a DFMR, but the filter itself and the mechanism of the backcheck valve provide the 2 levels of containment (Fig. 3, upper left).

For upload and initial stowage of the MCM, it was packed in a Ziplock bag to ensure the 3rd LOC.

For adding water and additives to the cement, standard 20 ml or 50 ml medical syringes were used. These syringes are very sturdy, but as per design of these syringes, the plunger can be removed from the barrel. To assure that the plunger stays in place a clamp was designed that connects the finger hold of the barrel with the top of the plunger (Fig. 1b). These syringe clamps were 3D printed from polycarbonate in different sizes to reflect the different amounts of liquid that were used for each experiment set. At the outlet of the syringe, a two-way valve was glued to the syringe followed by a 3 cm long piece of polyurethane tubing that was equipped with Luer Lock connectors at each end. This tube decouples the syringe from the concrete mixer and prevents leverage forces on the concrete mixer, which could lead to damage on the backcheck valve.

Some of the syringes contained air entraining agent that was classified as a toxic hazard level 1 substance, which requires two LOC. One LOC was provided by the syringe, and one by a simple plastic ziplock bag that contained an absorbent towel as save guard for leakage. To also ensure the two levels of containment after injection, a second ziplock bag was used.

During operations, the third level of containment was provided by the ISS Glovebag. This glove bag is a commercial item^28^ modified to be connected to the mobile work area on the space station. The samples could be inserted and removed via a zipper on one side of the glove bag.

### Hardware qualification and tests

**Fig. 4:**
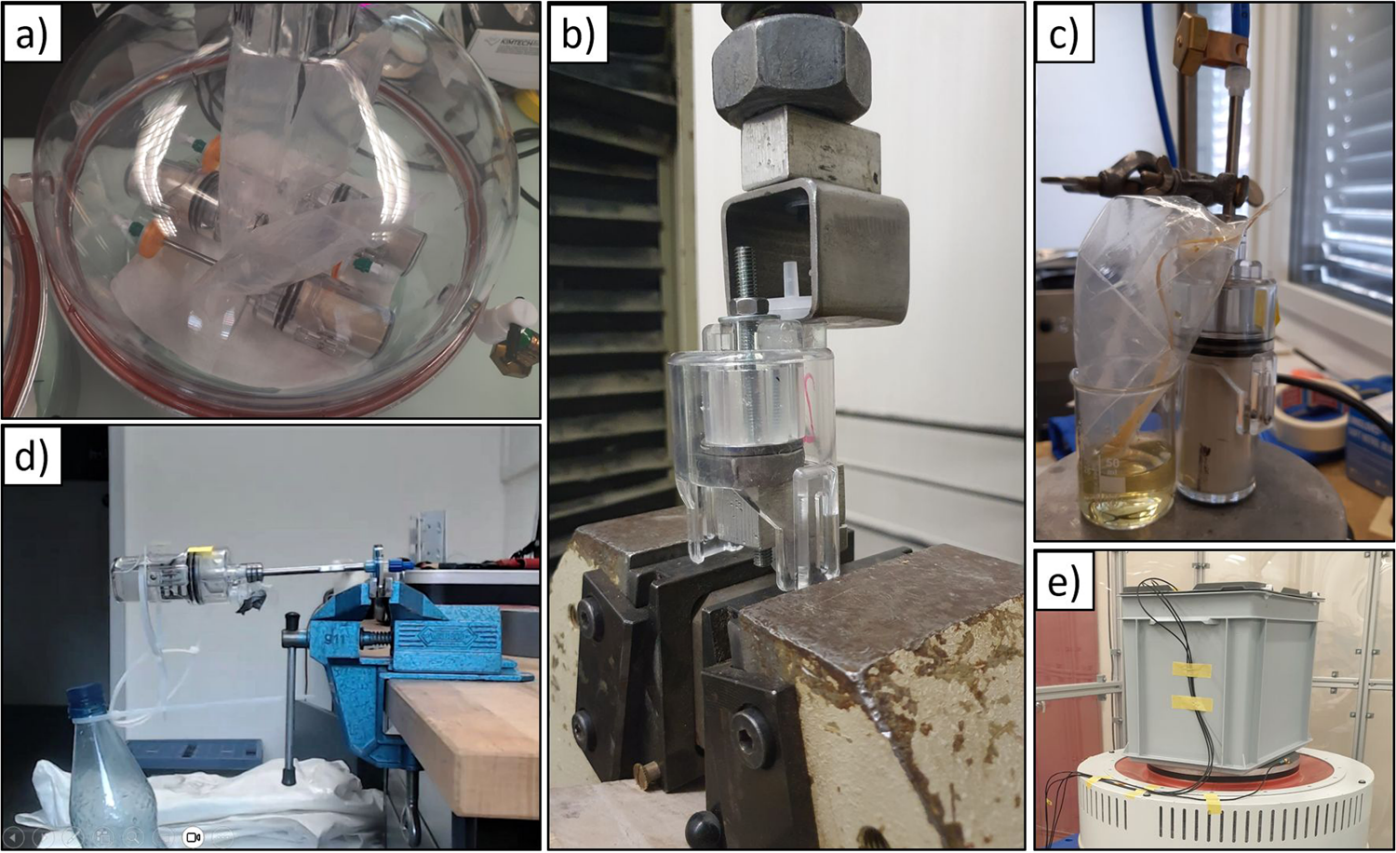
Qualification tests for the MCM. **a** vacuum leak test, **b** bonding test, **c** airflow test, **d** test of connection filter/tube, **e** vibration test.

Extensive tests and calculations were carried out to qualify the MASON Concrete Mixer for space flight and to verify the validity of the design:

At first, some prerequisites had to be clarified. The glues were qualified with the materials they should be used on by a pull test (Fig. 4b). Very good bonding was achieved between metal and polypropylene (PP), PP and methyl methacrylate-acrylonitrile-butadiene-styrene (MABS), MABS and MABS, PP and PP as well as polyethylene terephthalate (PET, Tritan MX 731) with PP using Loctite 4902^23^ and SF770 primer^29^. Bonding between polycarbonate (PC) and PP using Araldite 2015^27^ was unsatisfactory.

In addition, also the particle size of the cement powder had to be determined. The data sheets usually end with the detection of 0.5 µm particles, but it was unclear if smaller particles exist that might escape the mixer and invalidate the LOC concept with the 0.45 µm filter. Therefore, an airflow was established through a cement filled concrete mixer and the 0.45 µm filter (Fig. 4c). Detection of low levels of cement utilized the fact that cement, when wet, turns alkaline. Alkalinity can be proven on a pH paper or a solution with pH indicator. No release of particles could be found even when 500 l of air at a flow of 20 l/min were exhausted over the filter.

The MCM itself also had to undergo a variety of other tests, like a pressure leak test at 3.5 bar delta pressure (MDP), and a material compatibility test with the chemicals used. Here it was discovered that abrasion and elevated temperature produced by the hydratization of the concrete do not have an effect on the stability of the container. The cement powder did not have an influence on the container stability as well.

One other thing that needed to be tested were the loads the crew could induce on the concrete mixer, especially to the filter assembly on the mixing tube (Fig. 4d). The loads that were used for testing and qualification were according to NASA requirements documents^18^. In an earlier phase of the hardware development, a breaking of the interface between metal tube and the hydrophilic filter was experienced. This was solved by directly screwing the filter into the thread on the metal tube and potting the gap between the metal tube and the female Luer connector on the filter. This connection was especially tested for crew loads in a series of force tests on the hydrophilic filter. Impacts of the crank and bending forces of up to 3 Nm were sustained by the hydrophilic filter.

A verification for launch loads was performed as well. Nominal vibration tests were performed at 0 DB upright with combined Launcher Profile (Fig. 4e). The hardware integrity was ensured even under increased amplitude up to +12DB (100 g peak loads). The integrity was tested afterwards with a leak test.

As all tests were successfully carried out, the assembly of the flight hardware could be started at BIOTESC. In the assembly procedure, each individual step (a total of 118 steps for MCM, 36 for the syringes) was described in detail, documented, and checked by a qualified assurance. All parts and connections were inspected during and after assembly and each build MCM was leak tested at 350 mBar for 30 min (Fig. 4a), accepting only containers that had a weight loss of maximum 20 mg.

### Evaluation of MCMs feasibility for concrete specimen production

During the development and the qualification of the MCM, tests on the feasibility for concrete specimen production in the intended way were carried out continuously according to the protocol described in “Methods” section. These investigations included test on the demolding of the specimens as well as tests on the properties of the obtained specimens.

The concrete samples could be retrieved easily and efficiently from the MCM. The screws were first removed and then the retention and mixing chamber of the MCM could be separated with a little force. A small hole had to be drilled in the bottom of the mixing chamber so that a pressurized air nozzle could be attached there. Due to the slightly conical shape (0.5° inclination) and the smooth surface of the mixing chamber the concrete cylinder was pushed out with the compressed air force easily. During the EST, 64 MCM were used, and all the concrete samples could be retrieved without damage or any problems by this method. The ground test samples were examined in the same way as planned for the microgravity samples to verify the test methods and to show, that the concrete samples produced with the MCM are suitable to obtain scientific results.

Since no buoyancy or sedimentation is expected in the fresh concrete in microgravity, the samples produced in space will be examined primarily with regard to the air void and mass distribution as well as the effects on the mechanical properties, especially the compressive strength (see Table 2).

**Table 2:** Sample properties and test methods.

Description	Test method
Appearance, mass and density	Survey, dimensions, weight and density measurement
Air pore content and distribution	X-ray computed tomography (CT), optical microscopy and mercury intrusion porosimetry (MIP)
Characteristics of the microstructure	Scanning electron microscopy (SEM) and micro x-ray computed tomography (µ-CT)
Mechanical properties (compressive strength, stiffness and E-modulus)	Ultrasound measurement and destructive pressure test

In total, 20 different concrete mixtures were produced, containing different types of cement, aggregates, and additives. Selected mixtures are presented in the Supplementary Information.

To show that the concrete specimens produced in the MCM have similar and reproducible properties, some experimental results are presented here in extracts. The investigations on the properties of the specimens are still ongoing, the complete results will be published at a later point in time.

**Fig. 5:**
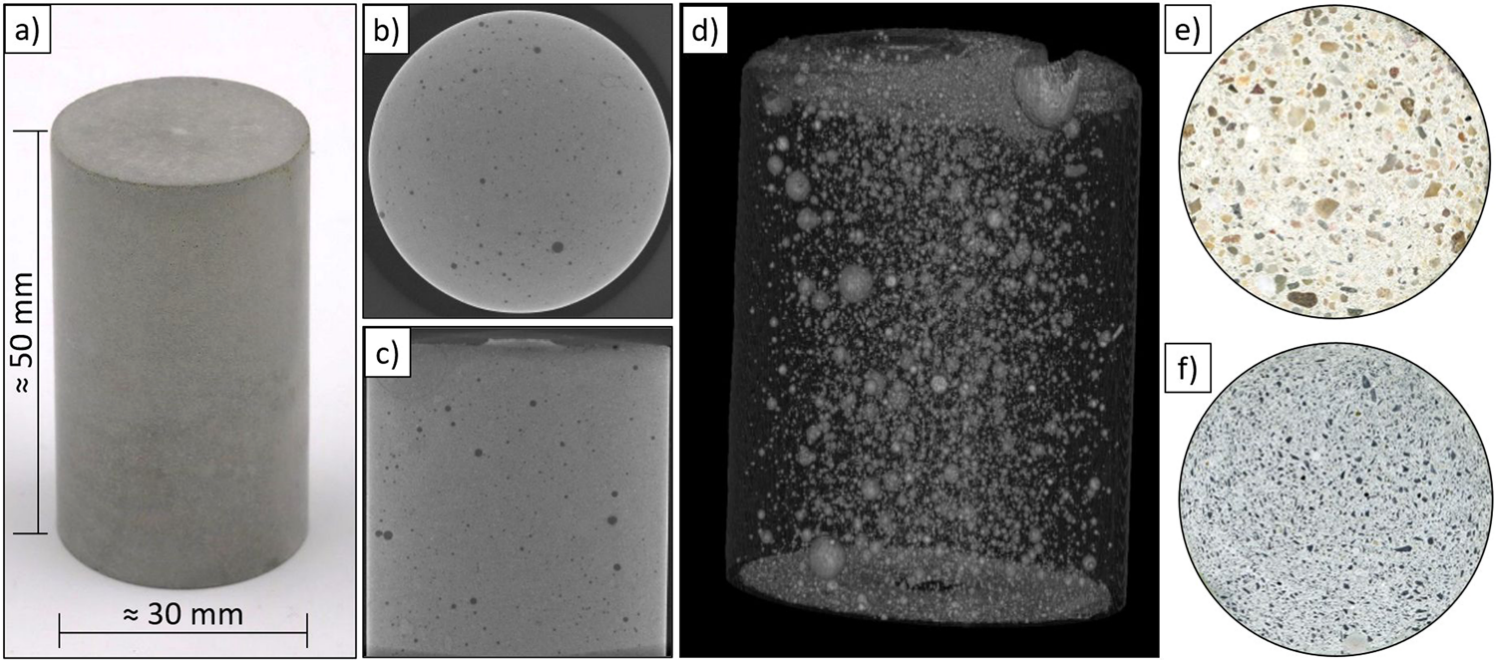
MCM samples. **a** shape, **b** cross and **c** longitudinal section (ct-scans), **d** 3d air pore distribution (ct-scan), **e** standard sand and **f** Regolith cross section scans.

First, it should be noted that the surfaces of all the specimens produced in the MCM were smooth, without voids and corresponded to an exposed concrete quality (see Fig. 5).

**Fig. 6:**
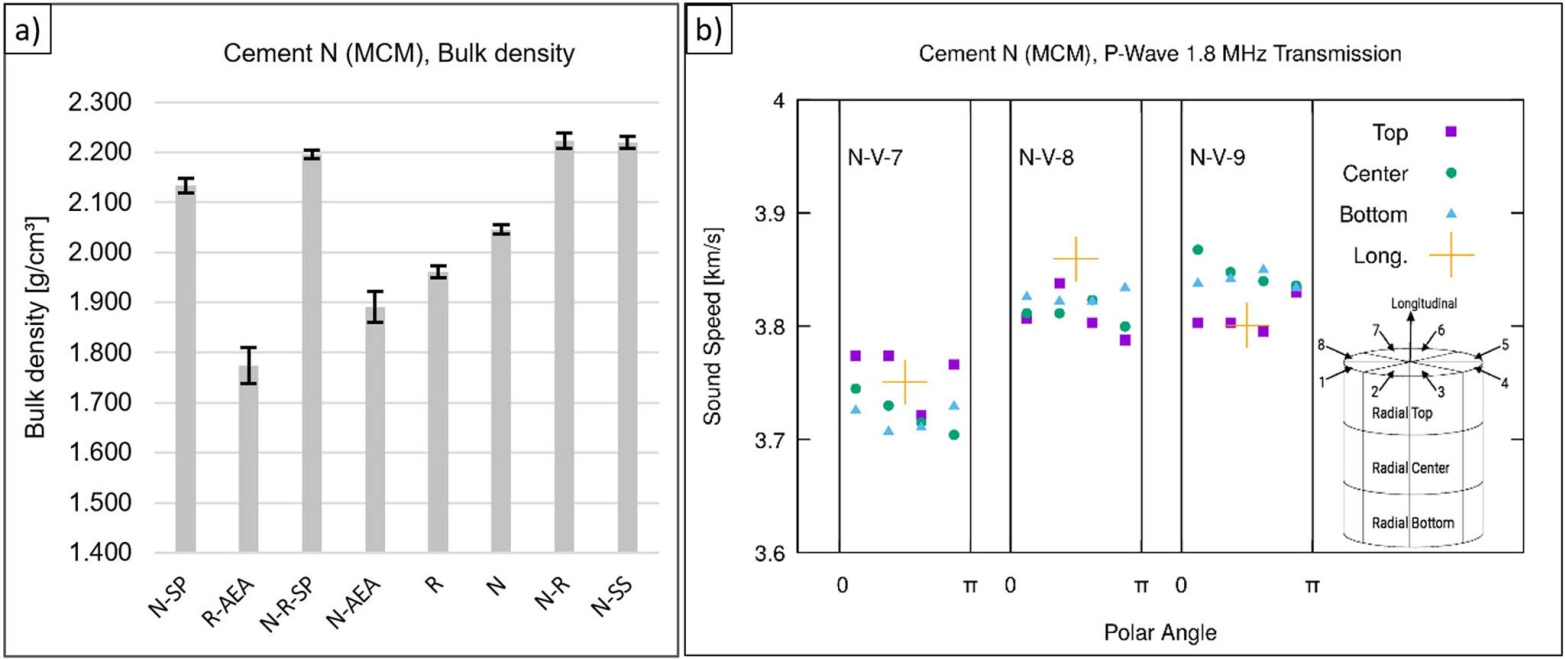
Bulk density and ultrasound results. **a** Bulk density and standard deviation of different MASON mixtures (24 samples produced in MCM; the error bars indicate the maximum and minimum of measured values from 3 individual measurements for each sample composition), **b** ultrasound results for samples of cement paste N (see Table 3), produced in the MCM in upright (vertical, indicated as N-V) orientation, for transmission directed either along the cylinder axis or radially, with opposite emitter-receiver directions identified and averaged.

Figure 6 shows the bulk density and standard deviation of different MASON mixes. It can be seen from the low standard deviation that the specimens have a uniform geometry and are homogeneously mixed. In detail, some results of cement paste samples are shown in Table 3. Furthermore, ultrasound transmission was measured based on the method presented by *Maruyama* and *Igarashi*^30^, but using 1.8 MHz P-wave pulses either in radial direction or along the cylinder axis. In radial direction, eight polar angles (modulo emitter-receiver reversal) in three regions were used (as sketched in inset of Fig. 6). Samples of the same mixture show consistent sound speed within 100 m/s and a dynamical Young’s modulus of 26.2 ± 0.7 GPa. Due to the natural composition of the cement slight variations in the results are normal even under optimal lab conditions. The MCM samples show good homogeneity and reproducibility, so that the good performance of the MCM can be confirmed.

**Table 3:** Comparison of sample N produced in MCM.

Example sample N		N-V-7	N-V-8	N-V-9
Diameter (geom. mean)	[mm]	29.87	29.87	29.90
Length	[mm]	46.90	46.65	46.55
Volume	[cm³]	32.85	32.68	32.69
Weight	[g]	66.03	65.84	66.71
Bulk density^a^	[g/cm³]	2.01	2.01	2.04
Sound Speed	[km/s]	3.73 ± 0.02	3.82 ± 0.02	3.83 ± 0.02
Young’s modulus^b^	[GPa]	25.1 ± 0.3	26.4 ± 0.3	26.9 ± 0.3

^a^Raw/Envelope density.

^b^Dynamic Young’s modulus based on ultrasonic pulse velocity method.

### On orbit operations

**Fig. 7:**
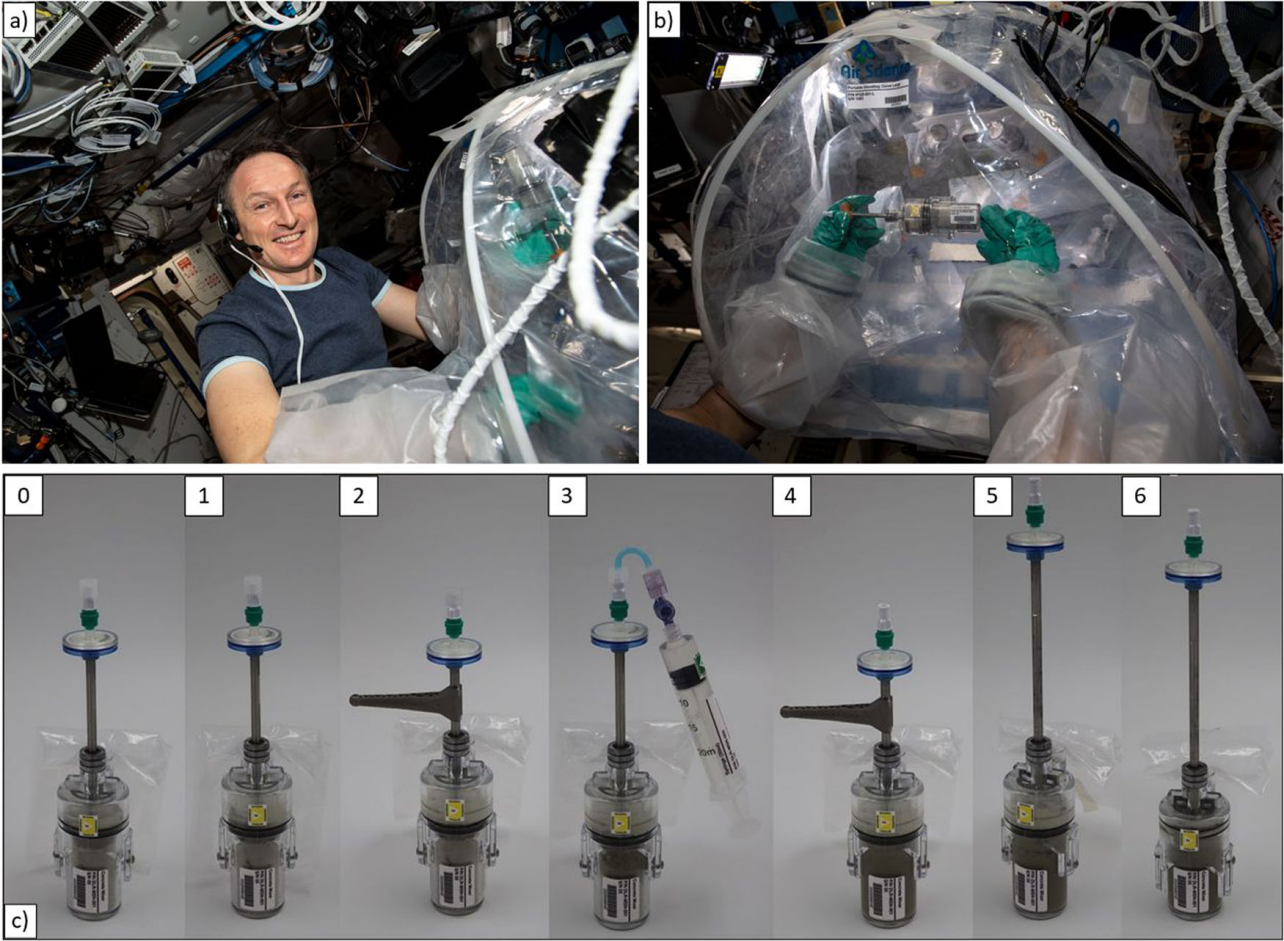
Handling of the MCM. **a**, **b** Use of the MCM on the ISS by Matthias Maurer during “Concrete Hardening” experiment [ESA/DLR], **c** major steps of the mixing process. The authors affirm that human research participants provided informed consent for publication of the images in Fig. 7.

The Concrete Hardening hardware was uploaded to the International Space Station with SpaceX CRS-24. The experiment was executed successfully inside the portable glove bag on ISS February 1st and 2nd 2022 by Matthias Maurer (Fig. 7), who was supported by the ESA User Support Center (USOC) BIOTESC. He reported back that the MCM performed well under ISS conditions in his hands. However, he made some suggestions to improve the design of the experiment: First, he proposed to choose a symmetric design of the crank for better handling. Second, he suggested to include a procedure to control the distribution of liquids, e. g. by centrifugation. A further optimization could be to remove air from the MCM by means of a vacuum pump.

The Concrete samples were left to harden on board until their return. The specimens safely returned to Earth with SpaceX CRS-25 (landed 20th August 2022) and are now in the hands of the MASON project team for intensive scientific investigations. On ground 223 MCM were used to produce identical concrete reference samples (120 in 1-g, 33 with RPM, 70 with clinostat). With EST and ISS experiment a total number of 351 MCM were in use for scientific analysis.

## Discussion

Within the MASON project a concrete mixer (MCM) was developed, tested, and qualified for use on the ISS. With this hardware concrete samples can be produced in microgravity by astronauts, which, due to the defined cylindrical shape, are ideally suited for later scientific analyses. The effects of microgravity on the properties of concrete as microstructure, air pore distribution, density, and compressive strength can be determined optimal, thereby providing a major contribution to future constructions in space. The MCM can be used very flexibly also for other two-component binders or construction materials and can be built in batch production fast, cheap and in large quantities. By means of first tentative tests on specimens produced using the MCM on ground, the general feasibility of the hardware in the intended way was proved. Finally, 64 concrete samples in MCMs were sent to the ISS, where the German ESA astronaut Matthias Maurer performed the experiment within the Cosmic Kiss mission in February 2022. On the ISS, the MCM performed well and allowed the execution of the experiment successful in terms of handling and safety. Based on his experience in µg, Matthias Maurer has suggested small improvements for the MCM to further enhance performance for future applications. Complete results of concrete sample properties and the feasibility of simulating microgravity during the solidification process using a clinostat and a random positioning machine will be presented after evaluation of the ISS and ground reference samples. In close communication with the American Penn State University and the designated NASA staff, future applications are already being considered, such as a moon mission.

## Methods

In total, 64 samples were processed by the crew on the ISS, in particular by ESA astronaut Matthias Maurer. Operation and handling, i.e. for storage transfer, involved ESA astronaut Samantha Cristoforetti. The authors affirm that human research participants provided informed consent for publication of the images in Fig. 7.

### Bonding

For bonding between Stainless Steel, MABS (Methylacylate-Acrylnitiril-Butadien-Styrol), Polycarbonate, Polypropylene and Tritan MX731 Loctite 4902 was used in combination with the primer Loctite SF770. Both surfaces were cleaned first with Isopropanol, then primed with Loctite SF770. Loctite 4902 was added on one surface and the parts were pressed together for 10 s and cured for 72 h at ambient temperature and humidity.

Bonding strength was measured by pulling the bonded pieces apart at a speed of 2 mm/min.

### Potting

The hydrophilic filter was potted onto the stainless steel tube, and the hydrophilic filter to the retention chamber and plastic bag with Araldite 2015. Curing was performed at 40 °C for 16 h at ambient humidity.

### Air flow test

An airflow of 20 l/min was established through the MCM for 25 min. The exhaust air was led over a pH indicator paper and in addition bubbled into a beaker containing 25 ml 100 mM NaCl solution and phenol red as pH indicator.

### Pressure tests

The MCM was assembled, and the hydrophobic filter was replaced by a cap to close the exhaust port. The MCM was filled with water. The syringes were filled with water and the syringe clamp was applied. A pressure of 3.5 bar was applied to the hardware under test for 15 min. The H/W was inspected for damage and loss of pressure over time.

### Vibration testing

Vibration testing of the Concrete Hardening hardware was performed at the Fachhochschule Nordwestschweiz (FHNW) on a TIRA TV57315 shaker. Launch loads for up to +12 dB were tested for Space X, Soyuz, Progress and Cygnus. After inspection, a pressure test and a vacuum leak test were performed to guarantee integrity of the H/W after vibration testing.

### Vacuum leak test

Concrete Mixers and syringes were subjected to a vacuum leak test. The samples were weight on an analytical balance (Mettler Toledo AE260) before and after the vacuum leak test. The H/W was inserted into a bell jar. Pressure was reduced to 350mbar for 30 min. H/W was inspected for visible leaks, weight and excluded if more than 20 mg of substance were lost.

### Chemical compatibility test

All items that can get in contact with cement powder were placed inside cement powder for 6 months. The items were cleaned and inspected.

Syringes were filled with 10% Air Entraining Agent or 20% Superplasticizer and stored for 6 months. The items were cleaned and inspected and subjected to a pressure leak test.

### Abrasion test

Influence of abrasion was tested by extensive mixing of three regolith samples for 10 min followed by inspection and a pressure test as described above.

### Mixing procedure

The major steps of the mixing procedure are shown in Fig. 7c and are explained in the following sequence:

1. Starting position of the MCM,
2. Pull up the retention chamber to increase space for mixing,
3. Attach MASON Crank, mix dry ingredients for 1 turn and detach MASON Crank,
4. Attach syringe, inject liquid, and remove syringe,
5. Attach MASON Crank and mix concrete for 2 min by rotating and moving the mixer,
6. Align mixer with the wings of the MCM, wiggle and pull up the mixer tube until blade is visible,
7. Push lower and upper part of the MCM together to reduce space and compress the concrete.

The whole process takes in average about 5 min crew time for each MCM.

Before space flight, the hardware, timing and on orbit scenario were tested on ground in an Experiment Sequence Test (EST) at the User Support Center BIOTESC in Hergiswil, Switzerland.

### Ultrasound transmission analysis

Waveforms were recorded using a digital oscilloscope (Picoscope 5000 series) and Picoscope 6 software. Using custom-written code, the waveforms were normalized and averaged over each pair of opposite receiver-transmitter orientations, then the time-of-flight was determined using a threshold of 0.04 relative to the signal maximum. In Fig. 6, results are plotted using gnuplot and the illustration showing the measurement directions and sub-regions within the sample is sketched using GIMP.

### X-ray computed tomography analysis

Tomograms were obtained by a General Electrics Nanotom 160 NF with 15 µm voxel size and initially analyzed using Avizo 9 software, providing the shown slices and 3D view in Fig. 5.

### Patents

After successful qualification of the MASON concrete mixer by the ESA safety panel, the design of the MCM was registered for patent approval^31^.

### Reporting summary

Further information on research design is available in the Nature Research Reporting Summary linked to this article.

## Supplementary information

Supplementary Information

Reporting Summary

## Data Availability

The data generated and analyzed during the current study are available upon reasonable request.
