# Supplementary material for: Space hardware for concrete sample production on ISS “MASON concrete mixer”
Source: NPJ Microgravity. 2023 Jul 21;9:57. doi: 10.1038/s41526-023-00304-0 (PMC10361957; doi:10.1038/s41526-023-00304-0)
Supplement: Supplementary file 1 — Supplementary Information [file 41526_2023_304_MOESM1_ESM.docx]

## Supplementary Information

Supplementary Table 1: Selected concrete mixtures produced with the MCM

| **Name** | **Cement** | **Aggregate** | **Additives** | **w/c** |
| --- | --- | --- | --- | --- |
| N | Ordinary Portland Cement (OPC)  CEM I 42.5 N | - | - | 0.35 |
| N-SS |  | CEN-Standard Sand (0/2) | - | 0.68 |
| N-R |  | Regolith EAC-1A (0/1) | - | 0.70 |
| N-SP |  | - | Superplasticizer | 0.29 |
| N-R-SP |  | Regolith EAC-1A (0/1) | Superplasticizer | 0.48 |
| N-AEA |  | - | Air Entraining Agent | 0.35 |
| R | OPC  CEM I 52.5 R | - | - | 0.40 |
| R-AEA |  | - | Air Entraining Agent | 0.39 |

## Supplementary figure legends

Supplementary Table 1: Selected concrete mixtures produced with the MCM
